# Physiologically Persistent *Corpora lutea* in Eurasian Lynx (*Lynx lynx*) – Longitudinal Ultrasound and Endocrine Examinations Intra-Vitam

**DOI:** 10.1371/journal.pone.0090469

**Published:** 2014-03-05

**Authors:** Johanna Painer, Katarina Jewgenow, Martin Dehnhard, Jon M. Arnemo, John D. C. Linnell, John Odden, Thomas B. Hildebrandt, Frank Goeritz

**Affiliations:** 1 Department Reproduction Management and Reproduction Biology, Leibniz Institute for Zoo and Wildlife Research, Forschungsverbund Berlin e.V., Berlin, Germany; 2 Department of Forestry and Wildlife Management, Faculty of Applied Ecology and Agricultural Sciences, Hedmark University College, Campus Evenstad, Elverum, Norway; 3 Department of Wildlife, Fish and Environmental Studies, Faculty of Forest Sciences, Swedish University of Agricultural Sciences, Umeå, Sweden; 4 Norwegian Institute for Nature Research, Trondheim, Norway; Federal University of Parana (UFPR)) – Campus Palotina, Brazil

## Abstract

Felids generally follow a poly-estrous reproductive strategy. Eurasian lynx (*Lynx lynx*) display a different pattern of reproductive cyclicity where physiologically persistent *corpora lutea* (CLs) induce a mono-estrous condition which results in highly seasonal reproduction. The present study was based around a sono-morphological and endocrine study of captive Eurasian lynx, and a control-study on free-ranging lynx. We verified that CLs persist after pregnancy and pseudo-pregnancy for at least a two-year period. We could show that lynx are able to enter estrus in the following year, while CLs from the previous years persisted in structure and only temporarily reduced their function for the period of estrus onset or birth, which is unique among felids. The almost constant luteal progesterone secretion (average of 5 ng/ml serum) seems to prevent folliculogenesis outside the breeding season and has converted a poly-estrous general felid cycle into a mono-estrous cycle specific for lynx. The hormonal regulation mechanism which causes lynx to have the longest CL lifespan amongst mammals remains unclear. The described non-felid like ovarian physiology appears to be a remarkably non-plastic system. The lynx's reproductive ability to adapt to environmental and anthropogenic changes needs further investigation.

## Introduction

The Eurasian lynx (*Lynx lynx*, *LINNAEUS 1758*, hereafter called “*lynx*”) is a large carnivore with a wide distribution in Eurasia, from the western Alps to the Russian Far East and from the southern Balkans to northern Scandinavia. In Europe they have undergone a historic period of decline, with some recovery in the most recent decades [1]. The spectrum of implied global change is raising many questions about the ability of wildlife to adapt to changes in their environment. A great deal of conservation and research focus is underway to try and understand the extent to which lynx can adapt to human modified landscapes [2] and impacts such as harvest [3]. Specific concerns regarding climate change and the way it may shape and influence seasonal patterns of resource availability and productivity are a cause for concern regarding many species. A major emerging topic is the extent to which species' phenology, especially the timing of reproduction, is able to respond to possible changes to keep pace with changes in seasonality [4], [5]. However, all species clearly show some constraints in their ability to adapt to change, as traits vary hugely in their reproductive plasticity. Understanding the physiological basis of the mechanisms that control reproduction is essential to understanding the potential for a species to adapt.

Many felid species are known to express a poly-estrous reproductive pattern: being able to mate several times a year [6]. In lynx, however, a “non-felid like” ovarian cycle [7] was recently documented which includes a mono-estrous cycle (only one estrus per year); speculated to be driven by an unusual reproductive feature – persistent *corpora lutea* (CLs) with a constant progesterone (P4) secretion. The assumption, that this may suppress the ovarian activity, and therefore create mono-estrous reproduction, was based on histological and endocrine examinations of lynx ovaries obtained from necropsies [8], [9], occasional ultrasound examinations of live animals and fecal hormone analyses [7], [10], [11]. To provide final proof that CLs remain active independently of pregnancy and lactation for more than one reproductive cycle, the same individuals need to be examined over a period of at least two cycles.

The ultrasound approach produces a high quality image for non-invasive soft tissue examinations and can be used to obtain clear information about the status of reproductive organs [12]. Thus, 3D ultrasound is being used more often for pregnancy diagnostics in wildlife medicine [13] and to study ovarian topography and function in various species [12], [14], [15]. Topographic maps of each ovary can be generated to demonstrate the exact position of individual CLs over time. Doppler color flow has already been used to quantify ovarian blood flow in lynx ovaries [16].

The present study used detailed longitudinal data of healthy lynx females held in zoos. The study includes the evaluation of the formation of CLs after ovulation in pregnant and non-pregnant animals, the luteal function during and after pregnancy or pseudo-pregnancy, as well as the luteal regression before next ovulation. To exclude that the results were an artifact of working with captive animals under artificial conditions, we took advantage of access to free-ranging lynx to conduct control examinations.

## Materials and Methods

### (a) Ethics statement

The examinations of captive lynx were performed when the animals were immobilized for other reasons, including veterinary monitoring, minor health intervention or due to captive animal management reasons. The methods applied, and the study-design, were in agreement with the animal ethics and welfare committee at the Leibniz Institute for Zoo and Wildlife Research (IZW, Berlin, Germany. No: 2010-01-01). The study of free-ranging lynx was conducted within the frames of the Scandinavian Lynx Project, Scandlynx (http://scandlynx.nina.no/). The free-ranging lynx were being captured for ecological studies related to demography and predator prey relationships [17] totally unrelated to this study. All capture and handling procedures were approved by the Norwegian Experimental Animal Ethics Committee and followed their ethical requirements for research on wild animals (permit numbers 2012/206992 and 2010/161554). In addition, permits to capture wild animals were provided by the Norwegian Directorate for Nature Management.

### (b) Animals

This study was conducted on ten captive female lynx examined 2-6 times (three animals were examined twice, one animal three times, two animals four times, three animals five times and one animal six times) each between April 2010 and July 2012. The reproductive history of each individual is listed in Table S1 in the electronic supplementary materials (ESM). The captive animals were housed in seven different zoological gardens within Germany (ESM, S1). They were all fed a standard zoo-carnivore diet. They were kept under various conditions; always solitary (N = 1), solitary for most of the year but then paired during the breeding season (N = 2), as mother-daughter groups (N = 2), as permanent female – male pairs (N = 2), or as family groups with last years' cubs and a male (N = 3). They were exposed to natural light and climates of 52°–53° N and 11°–13° E. The animals were on average 6 years old (range = 1.9–20 years) and weighed on average 17 kg (range = 12–19.5 kg). In feline species, pseudo-pregnancy is defined as a non-pregnant luteal phase after ovulation. Two of these animals were examined during pregnancy (days 7, 21 and 31 post copulation) and lactation (one examination each, 3 months post-delivery).

Ten free-ranging lynx were examined in February and March in northern Norway in 2011 and 2012 [17]. They were living in natural habitats with natural light and climate conditions ranging from 61°–73° N and 11°–17° E. They were on average 5 years old (range = 3–12 years) and weighed on average 17.7 kg (range = 16.8–18.4 kg). The free-ranging animals were documented to have a mean litter size of 2.2 [18].

### (c) Anesthesia and sampling

The captive lynx were darted inside their enclosures using a blowpipe and a 3 mL dart syringe equipped with a 22 gauge dart-needle (1.2×38 mm) (all from DanWild LLC, Dan-inject dart guns, TX, USA). An initial dose of 0.06 mg/kg Medetomidine (0.1%, Domitor, Orion Corporation, Espoo, Finland) plus 4.0 mg/kg Ketamine (Ketamine 10%; Essex GmbH, Munich, Germany) was used. During anesthesia, the animals were supplied with intranasal medical oxygen and intravenous isotonic NaCl-Infusion (0.9%, Braun, Tuttlingen, Germany). Respiration, heart rate, pulse-oxymetry (Nellcor, CA, USA), rectal temperature and eyelid-reflexes were constantly monitored.

The free-ranging lynx were darted from a helicopter and immobilized with medetomidine plus ketamine, using previously established protocols from Arnemo et al. [19], [20]

Blood was withdrawn from the free-ranging and captive lynx from the *vena cephalica* into serum and EDTA tubes (Sarstedt, Nürnbrecht, Germany). Vaginal smears, taken from the cranial region of the vagina, were obtained using a wet cotton swap. Cells were cropped on a glass slide, and fixed with a fixation spray (Roti-Fix, Carl Roth GmbH, Karlsruhe, Germany). The slides were stained following the Papanicolaou protocol (Papanicolaou Polychromlösung 3b, Carl Roth GmbH, Karlsruhe, Germany) and examined under a microscope (Jenaval, Carl Zeiss, Jena, Germany). Duration of anesthesia lasted on average 50 minutes (range = 45–70) and was reversed with an intramuscular dose of 5 mg atipamezole (Antisedan, Orion Corporation, Espoo, Finland) per 1 mg of medetomidine.

### (d) Ultrasound examinations of ovaries

In the captive lynx, small areas of fur were clipped in an area cranio-lateral to the second mammary complex (counted from caudal). Ethanol (70%) and ultrasound gel (Aquasonic 100, Parker Lab, NJ, USA) were poured on to achieve skin-contact for the ultrasound probe. An ultrasound laptop (Voluson i, GE-Health, Austria, Zipf) equipped with a 12 MHz linear probe (12 L – RS) and a 6–16 MHz volume probe (RSP 6 – 16 RS) were used for imaging. By applying brightness (B-) mode, the ovaries were visualized and length, width and breadth of the ovaries as well as maximum diameter of each CL were measured on screen. Volume was calculated using the mathematical formula “volume  =  pi * length * width * breadth / 4”. The three-dimensional mode (render and tomographic mode) was used to enumerate the CLs, to obtain a topographical map of each ovary, and to verify the individual CL's development and position. With the Doppler mode, the ovarian vessels were identified and ovarian vascularization was quantified by measuring the diameter of the *arteria ovarica* [16]. All examination steps were stored as videos to archive the data for post examination assessments. The software package 4D View (GE-Health, Austria, Zipf) was used to analyze the dataset.

Due to the harsh climate conditions (cold temperatures) and time constraints, only 2D ultrasound and Doppler modes were applied to the free-ranging lynx. The animals were not clipped; instead the fur was combed to reveal a small skin window.

### (e) Serum hormone analysis

Estrogens (E2), P4 and the prostaglandin F2α metabolite (PGFM) were analyzed in serum samples using in-house enzyme immunoassays [21], [22]. These assays have been previously validated for lynx [7], [21], [22]. The inter-assay coefficients of variation (CVs) when measuring PGFM in plasma samples were determined by repeated analyses of two pools containing 0.94 ng/mL and 3.3 ng/mL of PGFM and were 9.0 and 9.8%, respectively (n = 10). Intra-assay CVs are described in Finkenwirth et al. (2010) [22] (10.4 and 7.6%). Progesterone measurements in plasma samples revealed inter-assay coefficients of variation of 12.5% (n = 11) and 14.9% (n = 10), respectively. Intra-assay CVs are described in Dehnhard et al. (2010) [21] (9.0%). Estrogen measurements revealed an inter-assay coefficient of variation of 13.3% (n = 9). Intra-assay CVs are again described in Dehnhard et al. 2010 [21] (5.6%).

### (f) Statistical analysis

All tests were conducted using the software package R.2.14.1 [23]. A p-value of <0.05 was considered to be significant. We assessed correlations between different parameters (P4-, E2-, PGFM- serum hormone values, ovarian volume, CL number and size and the *arteria ovarica* diameter) using a Spearman rank correlation (rho, N_captive_  = 38 examinations, N_free-ranging_  = 10 examinations). In six captive lynx, structural and functional changes between reproductive stages (pro-estrus, estrus, met-estrus, prolonged di-estrus) were analyzed using a Quade-test for repeated measurements. In these animals examinations of all consecutive reproductive stages were possible. We compared differences during the months of February and March between the free-ranging population (N = 10 examinations) and the captive population (N = 10 examinations) using the Wilcoxon test (for non-normally distributed parameters) and the two samples t-test (for normally distributed parameters). For this analysis only one examination per individual was taken. All data are shared publicly in the ESM (Table S2).

## Results

### (a) Reproductive stages in lynx

**Figure 1:**
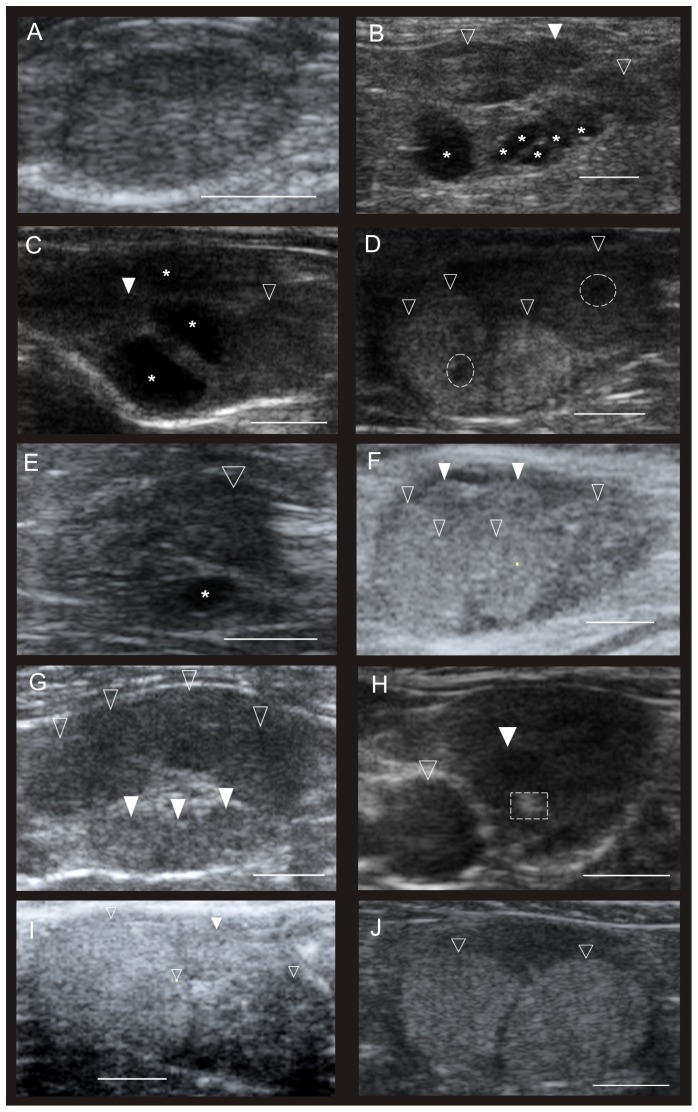
Ultrasonographical images of lynx ovaries during different reproductive stages. The ultrasound images (in b-mode) of lynx ovaries demonstrate different stages of ovarian activity. The white scale-bar indicates 1 cm. Asterisk indicates follicles, empty arrows show new CLs; full arrows show old *corpora lutea* (CLs) from previous years cycles. A: Juvenile lynx before puberty. Only the ovarian cortex is visible, no follicular or luteal structures; B: Pro-estrus in an adult lynx, small and medium sized, immature follicles appear next to CLs from previous years' cycles. C: Estrus in an adult lynx. Large, mature follicles and smaller immature follicles and an old CL from last year's cycle are visible; D: Met-estrus in adult lynx after recent ovulation. One old CL is visible. Two freshly ovulated, plicated follicles whose walls have begun to luteinize and formatting a new CL, centre parts are not yet fully closed (interrupted circles); E: second follicular wave, observed in only one animal during May, about 65 days after first estrus. Only one medium sized follicle visible; F: Pseudo-pregnancy in lynx. CLs without mating, no pregnancy, 1 month after spontaneous ovulation; G: Prolonged di-estrus during winter. Physiologically persisting CLs are hypo-echoic and still quite large; H: *Corpus albicans*. Old CL (interrupted rectangular) undergoes structural regression. CLs' tissue becomes denser and hyper echoic in ultrasound; I: Pregnancy at day 31 post copulation, CL appear hyper echoic; J: CL during lactation in lynx. Large, hyper echoic CL's.

**Figure 2:**
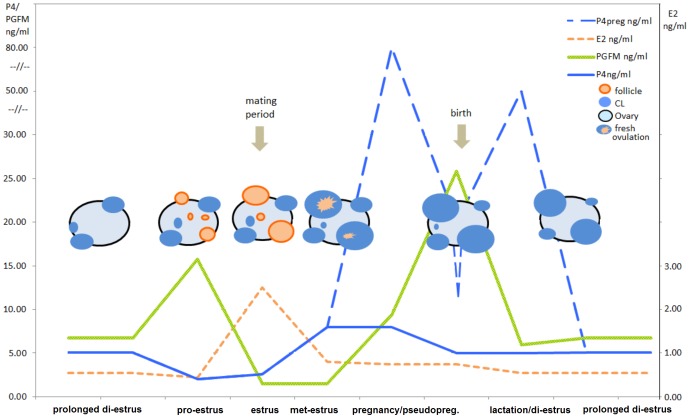
Schematic lynx ovarian cycle. Schematic diagram of the development of follicles and CLs within a reproductive year in pregnant and non-pregnant lynx. P4, E2, PGFM in ng/mL are shown with lines.

The different reproductive stages in female lynx were classified as pro-estrus, estrus, met-estrus, pregnancy, pseudo-pregnancy, lactation and prolonged di-estrus (Fig. 1; Table 1). Figure 2 summarizes the luteogenesis and luteal regression in a schematic. There were no biologically relevant, and significant differences between free-ranging and captive lynx during February and March 2011 and 2012 (please also see Table S3, ESM; Wilcoxon rank test: P4: W = 30, p = 0.86; diameter *a. ovarica*: W = 105, p = 0.07; ovarian volume: W = 172, p = 0.46; CL tissue: W = 129, p = 0.06; age: W = 112.5, p = 0.05 and Two sample t - test: E2: t = 0.11, p = 0.91, df = 16; PGFM: t = −1.42, p = 0.18, df = 12; number CLs: t = −2.17, p = 0.04, df = 35.5).

**Table 1:** Reproductive parameters in different reproductive stages.

cycle					CL per	CL tissue	ovarian	diameter.	number
stage	N	E2	P4	PGFM	ovary	(cm^2^)	volume	A *ovarica*	follicles
**pro-estrus**	10	0.59±0.34	2.65±2.77	2.59±0.81	3.26±1.69	0.88±0.64	2.18±1.44	0.22±0.04	1.53±2.43
**estrus**	4	1.49±0.04	2.08±0.70	1.23±0.45	2.50±2.17	0.78±0.62	1.55±1.05	0.24±0.00	1.83±1.72
**met-estrus**	3	0.78±0.86	13.07±8.1	1.46±0.44	2.17±2.32	1.02±1.61	1.93±1.19	0.23±0.08	1.00±1.26
**prolonged**	26	0.32±0.21	4.68±3.45	1.82±0.91	2.66±1.43	0.65±0.48	1.74±1.03	0.20±0.05	0.37±0.76
**di-estrus**									
**pregnancy**	3	0.56±0.14	84.05	2.61±1.18	4.17±1.17	1.55±0.66	3.80±2.33	0.29±0.05	0.67±1.21
			±83.85						
**lactation**	2	0.18–0.44	3.14–	4.41–1.48	2.75±0.50	1.66±0.92	2.35±0.96	0.25±0.03	0.00±0.00
			170.38						

Mean values ± S.D. of various ovarian and serum parameters in free-ranging (N = 10) and captive (N = 38) lynx examinations during the various stages of the reproductive cycle, based on a 2 year study (2010–2012). E2  =  serum estrogens in ng/mL, P4  =  serum progesterone in ng/mL, PGFM  =  serum prostaglandin F2alpha in ng/mlL, CL  =  *corpus luteum*, A. ovarica  =  *arteria ovarica*.

One captive female was a juvenile (19 month); accordingly we observed small ovaries (0.54 cm^3^ and 0.10 cm^3^) without follicular or luteal activity (Figure 1A). A good differentiation between cortical and medullar parts of the ovary was detected and hormone analyses revealed low E2 (0.16 ng/mL), and non-detectable P4 values.

Pro-estrus was found in lynx examined just before the mating season in early spring. These females had already undergone at least one reproductive cycle in a previous year (N_captive_  = 5, N_free-ranging_  = 5). Several hypo-echoic CLs were detectable on the ovaries using ultrasound (Fig. 1B). They exhibited various sizes encompassing 0.2–0.65 cm in diameter. The number of CLs (S = 26929.36, p = 0.002, rho = 0.38) and the level of P4 (S = 27142.29, p = 0.012, rho = 0.32) increased significantly with age. Vaginal smears collected from pro-estrus stages showed a typical image of primarily round to oval shaped, basophilic basal and intermediary cells or if already under higher estrogen influence they were with enlarged basophilic or acidophilic superficial cells (ESM, Fig. S1-A; cell proportions on average ± SD: parabasal cells (pbc)  = 17±16%, intermediate cells (imc)  = 46±8%, superficial cells (sfc)  = 35±17%, superficial anucleated cells (sac)  = 2±1%). The hormones were characterized by increased PGFM and a slight decrease of P4 (Table 1).

In one captive female a second behavioral estrus was observed by the keepers about 2 months after her normal seasonal estrus. This female was kept without a male throughout. During ultrasound examination one rather small follicle was seen in each of her ovaries (0.27 and 0.34 cm diameter, Fig. 1E). The corresponding hormone levels were not indicative of an estrus. Vaginal cytology showed intermediary and basophilic and acidophilic superficial cells (pbc = 27%, imc = 52%, sfc = 21%, sac = 0%). Her estrous behavior did not reach the same strength as during a proper estrus.

Estrus occurred at the end of February until mid-March for the captive lynx (N = 2) and at the end of March for the free-ranging lynx (N = 2). Behavioral signs of estrus (calling, rolling) lasted between 5 and 10 days for the captive animals observed in this study. Nevertheless, the zoo-keepers occasionally observed calling from the males or females already 3–4 weeks in advance. One captive female was in her first estrus. In this case we observed one follicle on each ovary (0.96 and 0.78 cm, Fig. 1B). The corresponding hormone concentrations were 1.58 ng/mL (E2), 1.58 ng/mL (P4), and 0.91 ng/mL PGFM. Estrous stage in older female lynx was characterized by a simultaneous appearance of follicles (0.01–0.95 cm diameter) together with hypo-echoic CL (Fig. 1C). In most cases, follicles were forming on both ovaries. Vaginal smears showed large superficial cells which were mainly acidophilic, with or without nuclei and partly cornified and folded (ESM, Fig. S1-B; pbc = 0±0%, imc = 1±2%, sfc = 49±40%, sac = 50±38%), the elevated E2 levels also pointed towards an estrous stage (Table 1).

Met-estrus, thus the period shortly after ovulation, was detected in three animals with clearly depicted ovulation scars (Fig. 1D, N_captive_  = 2, N_free-ranging_  = 1). Vaginal cytology showed mainly nucleus-free, acidophilic superficial cells (ESM, Fig. S1-C; pbc = 13±15%, imc = 16±6%, sfc = 29±9%, sac = 42±10%) and hormone levels indicative of luteal formation with elevated P4 levels (Table 1).

After the post-estrus luteal formation period, freshly formed fully functional CLs were judged to be a clear indication of a recent ovulation. The fresh CLs differed in their sono-morphology from the old CLs of previous cycles (Fig. 1F). They were not only bigger but also hyper-echoic. In general, the formation of new CLs was detected in both, the absence (N = 7 females) and presence (N = 3 females) of a mating partner and resulted in pseudo-pregnancy and pregnancy, respectively. In our study the ovulation rate was 100% each year in all females.

In pregnant females the secretion of P4 increased rapidly after ovulation. The CL tissues appeared hyper-echoic compared to pseudo-pregnant new CLs at the same time (Fig. 1I). These CL *graviditatis* were the same size as pseudo-pregnant new CLs (average 0.86 cm, range = 0.71–1.37 cm diameter) and they persisted for at least the 2 years of this study. During pregnancy P4 values were drastically elevated (>10 x), whereas in the pseudo-pregnant females (approx. up to 5 weeks post estrus) the P4 values were in the range obtained outside the breeding season. Five litters were born in captivity during the study period (4 triplets and 1 single kitten litters). During lactation the CLs (N_captive_  = 2, Fig. 1J) appeared to be hyper-echoic compared to pseudo-pregnant (Fig. 1G) females at the same time. Gestation periods with observed mating and birthing dates in the captive lynx were 66–70 days, since females were often mated over 2–3 consecutive days. Vaginal smears mainly showed acidophilic superficial cells or intermediate cells (ESM, Fig. S1-D; pbc = 7±9%, imc = 31±25%, sfc = 38±21%, sac = 24±29%). Hormone levels were different between the two examined females, with one female expressing very high P4 (170 ng/mL) in contrast to 3.1 ng/mL in the other individual.

After weaning (day 100 postpartum) the CLs appeared similar to CLs of non-pregnant females (N_captive_  = 24, N_free-ranging_ = 2) at the same time (Fig. 1G), and likewise hormone levels were not different. New CLs derived from this years' ovulation and old CLs derived from previous years' cycles (Table 1) were observed next to each other. They could be clearly distinguished by size (0.8–1.2 cm in diameter in new CL versus 0.2–0.65 cm for old CLs) and sono-morphologic texture (new CLs had a hyper-echoic appearance). The functional luteal activity outside the breeding season is hereafter referred to as prolonged di-estrus, or the phase of physiologically persistent CLs.

**Figure 3:**
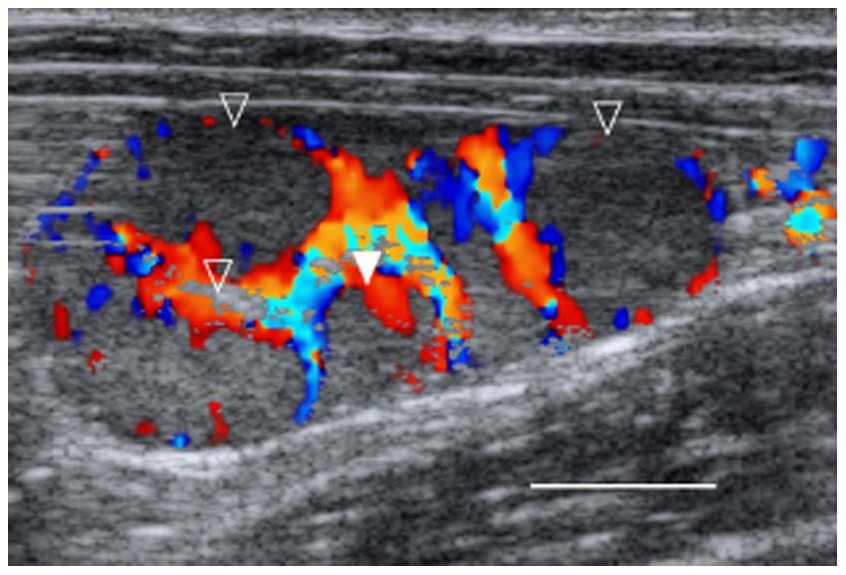
Persistent *corpora lutea* (CLs) of lynx in ultrasonography. Prolonged di-estrus in lynx outside breeding season in December. Empty triangle points at CLs from this years' ovarian cycle. Full triangle points out one old CL, at least two years old.

Values of P4 during prolonged di-estrus were significantly correlated with the intensity of vascular support measured using the diameter of the *A. ovarica* (Fig. 3, Spearman rank correlation, S = 10608.26, p<0.001, rho = 0.67), as well as with the number of CLs per ovary (S = 37774.55, p-value<0.001, rho = 0.39). Interestingly, no correlation was found either between month of the year and P4 concentrations in serum (S = 42362.31, p = 0.813, rho = 0.03), or to the intensity of vascular support (S = 42540.79, p = 0.164, rho = −0.18), when excluding estrus and pregnancy. Furthermore we found a positive correlation throughout the year between P4 and E2 in serum (S = 32824.11, p-value = 0.047, rho = 0.24) when excluding estrus. Vaginal cytology during prolonged di-estrus was variable; dominated either by basophilic basal cells, parabasal cells, or intermediate cells (ESM, Fig. S1-E; pbc = 25±24%, imc = 29±18%, sfc = 45±30%, sac = 1±1%). Repeated measurement within the same individual revealed a slow structural regression of CLs (diminishing diameter, decreasing echogenity) over time. A minimum life-span of two years was monitored in this study. The texture of regressing and cicatrized CLs in the final stage (*corpora albicans*) again appeared to be very hyper-echoic (Fig. 1H).

One captive animal was examined after its reproductive senescence (at 19 and 20 years of age). Steroid hormones measured were at very low levels with E2 = 0.39 ng/mL and non-detectable P4. Both ovaries appeared similar to juvenile ovaries, non-active and without functional bodies and only a minor vascularization.

### (b) Repeated examination of animals

Repeated examinations were performed in six captive animals encompassing all non-pregnant cycle stages (pro-estrus, estrus, met-estrus, prolonged di-estrus). No significant differences emerged when comparing the concentrations of serum P4, E2, PGFM, ovarian volume, the intensity of vascular support (diameter A. *ovarica*) number of CL and size of CL tissue in their different reproductive stages, excluding estrus (also see table S4 in the ESM; Quade – Test, df = 22, P4: F = 0.07, p = 0.93; E2: F = 2.26, p = 0.13; PGFM: F = 0.02, p = 0.98; ovarian volume: F = 0.21, p = 0.81; diameter A. *ovarica*: F = 0.88, p = 0.43; number CL: F = 0.61, p = 0.55; size of CL: F = 0.83, p = 0.45). The number of follicles was significantly different in estrus (Quade-Test, df = 33, number follicles: F = 3.61, p = 0.02) compared to the other reproductive stages, but not significantly different within the three stages outside estrus (Quade – Test, df = 22, F = 0.08, p = 0.92). A simultaneous rise or decline of P4 and E2 was noted outside the mating season. Furthermore the number of CL, the size of CL tissue, the size of ovaries, and the position of the CLs were verified in-vivo with a sonographical 3D topography map.

## Discussion

By repeated ultrasound and hormonal examination in captive lynx, our study confirmed the physiological persistence of CLs derived from ovulations (Fig. 2). The results were supported by opportunistic examinations of free-ranging lynx. The life span of lynx CLs lasted for at least two years (duration of this study). In contrast to other feline species CLs in lynx did not disappear after pregnancy or the assumed luteal phase of pseudo-pregnancy, which is supposed to last for two thirds of the pregnancy [11]. The lynx CLs experienced only a gradual loss in size over time, while they were still expressing an active vascularization [16] and a moderate luteal P4 production [8]. During the fecund years, no typical anestrus (complete ovarian inactivity) was observed indicating that lynx have evolved a different strategy than other felids. Furthermore, the functional lifespan of lynx CLs exceeds the lifespan of elephant's CLs, which were previously reported as the longest ever documented CL lifespan among mammalian species [15].

The lynx reproductive cycle shows new and so far unknown dynamics in luteogenesis and luteal regression amongst felid species (Fig. 2). Plication of the new ovulated follicle wall and luteogenesis was observed at the start of met-estrus (Fig. 1D). Freshly formed CLs co-existed next to the CLs from previous cycles. The freshly ovulated CLs produced higher steroid amounts during met-estrus and pregnancy with approximately 10- and 100-fold higher intra-luteal P4 and E2 levels respectively [8] and 10-fold increased serum P4 levels (this study). These “new” CLs persisted after birth or pseudo-pregnancy without shutting down their luteal function. This was indicated by steady P4 serum levels, and the positive correlation between serum P4 and the intensity of ovarian vascularization. The physiological persistence was also confirmed by a positive correlation between the number of CLs per ovary and the animal's age, indicating a long CL lifespan and a temporary accumulation of CLs on lynx ovaries. This is very atypical for felids where the luteal activity normally starts to regress after birth in pregnant, and after the end of pseudo-pregnancy in non-pregnant, females. At least in the domestic cat it is known that serum P4 drops below 1 ng/mL [6] after parturition, and the CLs structurally regress to *corpora albicantia* [24]. In lynx, serum P4 levels remained elevated at average (basal) levels of around 5 ng/mL. In contrast to P4, serum PGFM reflected different hormonal functions of persistent CLs. Serum PGFM was elevated during pro-estrus and late pregnancy (PGFM in serum free-ranging lynx, Scandlynx; mean  = 18.16 ng/mL, range  = 11.88–56.06, N = 6) which lead to a Prostaglandin F2α (PGF2α) mediated functional luteal regression (P4 decreased), without structural regression. Prostaglandins are known for their luteolytic effects, which decrease luteal blood supply, P4 secretion, and degrade CL structure [9], [25]. The elevated serum PGFM levels were in accordance with our previous study investigating intra-luteal prostaglandin concentrations in lynx. These intra-luteal prostaglandins were only elevated prior to the breeding season [9]. However, the pro-estrus elevation of serum and intra-luteal prostaglandins could not be mirrored by the levels of PGFM determined in urine or feces of lynxes [22]. This may indicate that PGF2α is only produced at moderate levels prior to the onset of estrus (in contrast to late pregnancy) to act as an intra-ovarian signal for a transitory functional luteolysis of persistent CLs, which may serve as a necessary prerequisite for follicular growth and ovulation. Experiments in domestic cats examining the effect of PGF2α have also shown that rapid and complete recovery of luteal function is possible [26] and that PGF2α does not always initiate structural luteal regression in cats either[25].

In the past, all felids were believed to be exclusively coitus induced ovulators. However, later studies have shown that some cat species are able to ovulate spontaneously. Most felids express a combination of induced and spontaneous ovulation with individual variability [11]. Our data on captive lynx clearly indicate that lynx are able to ovulate spontaneously. As shown by the appearance of new CLs after breeding season, all captive lynx ovulated each year, either after natural mating (4 studied cycles) or spontaneously in the absence of mating (13 studied cycles), without there being a detectable difference in the lifecycle of the CLs. Felids show a broad variety of reproductive cyclicity, mostly dependent on photoperiod; ranging from being poly-estrous and seasonal (e.g. *Felis silvestris catus*, *Panthera tigris*, *Panthera uncia*) to being poly-estrous and aseasonal (e.g. *Panthera leo*, *Panthera pardus*, *Puma concolor*) [11]. In contrast, all members of the genus *Lynx*, except the bobcat [27], are strictly seasonal breeders [7], [21], [28].

Henriksen et al. [29] presented a mean birth date in 150 litters from captive lynx and in 23 litters from wild Scandinavian lynx: 50% of the captive litters were born within a 13 day period, and within an 8 day period in wild animals. This is corroborated by our study and also the findings of Kvam et al. [30], showing that breeding periods and therefore birthing dates are all within a very narrow window, minor latitudinal differences aside. A second estrus and later parturition seem to be very rare, but possible under extraordinary conditions (abortion, resorption, early loss of offspring) [31]. In our study, only one captive female lynx showed muted behavioral signs of a second estrus, however, minor folliculogenesis in ultrasound images and slight E2 elevations, indicated that the second “estrus” was not fully expressed.

During the lifespan of an animal there is a limited period for fecundity. This period usually starts at puberty (in female lynx mostly in their second year [18] and lasts until death. A period of reproductive senescence, as we have found in one old female lynx, usually occurs only in captivity because free-ranging lynx normally die before reaching reproductive senescence [32]. The most frequently observed physiological mechanisms to time births in mammals are either limiting the period of fertility (seasonal breeding, e.g. Pallas' cat [11]) or extending the duration of pregnancy (delayed implantations, e.g.: roe deer, some mustelidae [33], [34]). Lynx seem to have developed a quite remarkable alternative strategy, where they limit the period of fertility by converting a poly-estrous cycle into a mono-estrous cycle. By doing this, they extend the duration of functional pseudo-pregnancy (luteal activity after ovulation without a pregnancy) for a prolonged time period to prevent follicular development before the next breeding season.

The hormonal support mechanism of persistent CLs still needs to be identified. In the domestic cat, prolactin is known to be a luteotrophic factor [35]. It might be suggested that the photoperiod response system acts via melatonin on the prolactin secretion, as shown for several seasonal breeders [36]. Up until now, only the involvement of intra-luteal (locally) produced prostaglandins can be suggested [9]. The source and neuro-endocrine regulation of prostaglandin secretion during pro-estrus in lynx remains unclear.

The evolution of a mono-estrous reproductive system in lynx could have primary (the ancestors of felids were mono-estrous) or secondary (adaptive) origins. A phylogenetic constraint, however, seems unlikely as mono-estrous breeding is a very unusual pattern among felids in general, and the most primitive member of the genus *Lynx*, the bobcat [37], is poly-estrous [27]. The alternative explanation is that the mono-estrous strategy has evolved as an adaption in order to time birth within specific times of the year. Interestingly we know from other felids that the body size, the period of parental care, and its habitat seem to be associated with what kind of reproductive pattern they may express [38]. Smaller wild felids (*Felis chaus*, *Felis silvestris* and *Felis serval*) reproduce twice a year, while larger wild felids (genus *Panthera*) in general only reproduce every other year [39]. Most medium sized wild felids give birth to only one litter per year. Unlike the lynx, other felids are in anestrus (no hormone activity) in between their annual breeding period, which may differ in length [38]. Female lynx investing early in maturation, ovulation and placentation have been shown to be significantly lighter in the following year, indicative of high costs of reproduction [40]. Yet, the costs of reproduction are not well understood in solitary, large carnivores, like the lynx. In lynx populations that depend on ungulates like roe deer there is no obvious reason to time birth to early summer as prey is more easily located and caught in winter, and neonates do not represent a major proportion of the diet [41]. For lynx that feed on smaller prey it is unclear as to whether there is a benefit to giving birth in early summer as prey density tends to fluctuate in multi-year rather than seasonal cycles [42]. Avoiding inclement weather, detrimental for neonates left alone for prolonged periods while the mother hunts, may be a factor that would argue against extremely early births, but would not hinder later births during mid to late summer and autumn, which would be associated with poly-estrous breeding. One potential explanation could view this as an anti-infanticide strategy. Infanticide by males is very common in many felids, such as pumas [43] as loss of kittens leads to a rapid onset of a second estrus, but a second estrus is a very unlikely event in lynx. In contrast, mono-estrous breeding, especially in a species that only use one year to raise kittens [44], would effectively remove any benefits from males killing kittens. Interestingly, there is no documented case of infanticide among wild Eurasian lynx [32], to our knowledge.

The lynx reproductive cycle seems to be a rather non-plastic system. We found no difference in reproductive pattern between either wild and captive lynx or between central and northern European populations. The only anticipated difference was found concerning the timing of breeding seasons, which is most likely latitude, and therefore delayed photoperiod, dependent. However, the captive and free-ranging populations both showed the same reproductive strategy i.e. being mono-estrous due to physiologically persistent, constantly P4 secreting CLs, without plasticity regarding this phenomenon.

The main conservation implications of these results is the confirmation that lynx are indeed mono-estrous. The fact that ovulation appears spontaneously implies that pregnancy is very dependent on having access to a male during the crucial period of estrus. Anything that reduces access to males in this narrow window would result in an entire year's reproduction being lost. Many of the larger lynx populations are subject to hunter harvest in some form, and hunting is normally conducted in the late winter because of the hunter's dependence on good snow conditions [45]. Lynx hunting is male biased [18] and there is therefore a very real chance that lack of access to males could reduce population growth to a greater extent than has been appreciated. The actual impact will depend on issues such as female behavioral responses to the lack of a male and on the ability of transient (non-territorial) males to fertilize females.

A second conservation implication concerns the ability of lynx to adapt to environmental changes. The physiology described in this paper indicates that lynx will have relatively little ability to adjust birth dates as it appears to be a remarkably non-plastic system. However, the full implications of this are not clear, because we do not yet know the environmental cues for the timing of ovulation in lynx.

## Supporting Information

Figure S1 **Vaginal cytology in lynx.** Vaginal cytology, stained with papanicolou at (A) pro-estrus, (B) estrus, (C) met-estrus, (D) pregnancy and (E) prolonged di-estrus stages. Black bar indicates 50 µm.
(DOC)

Table S1 **Origins and reproductive status of study animals.** The origins of the captive and free-ranging animals are listed below. Furthermore the reproductive period they have been examined is listed in the table.
(DOC)

Table S2 **Data sharing.**
(DOC)

Table S3 **Comparison between free-ranging and captive lynx.** Comparison of various ovarian and serum parameters between single examinations of free-ranging (N = 10) and captive lynx (N = 10) during February and March 2011 and 2012.
(DOCX)

Table S4 **Repeated measurements from six captive Eurasian lynx throughout one reproductive year (February – February).** Measurements were categorized in 3 time periods, excluding estrus (pro-estrus, pseudo-pregnancy, prolonged di-estrus) and tested if there are seasonal changes within an individual.
(DOC)
