# Supplementary material for: Physiologically Persistent Corpora lutea in Eurasian Lynx (Lynx lynx) – Longitudinal Ultrasound and Endocrine Examinations Intra-Vitam
Source: PLoS One. 2014 Mar 5;9(3):e90469. doi: 10.1371/journal.pone.0090469 (PMC3943960; doi:10.1371/journal.pone.0090469)
Supplement: Figure S1 — Vaginal cytology in lynx. Vaginal cytology, stained with papanicolou at (A) pro-estrus, (B) estrus, (C) met-estrus, (D) pregnancy and (E) prolonged di-estrus stages. Black bar indicates 50 µm. (DOC) [file pone.0090469.s001.doc]

**Figure S1: Vaginal cytology in lynx.** Vaginal cytology, stained with papanicolou at (A) pro-estrus, (B) estrus, (C) met-estrus, (D) pregnancy and (E) prolonged di-estrus stages. Black bar indicates 50µm.


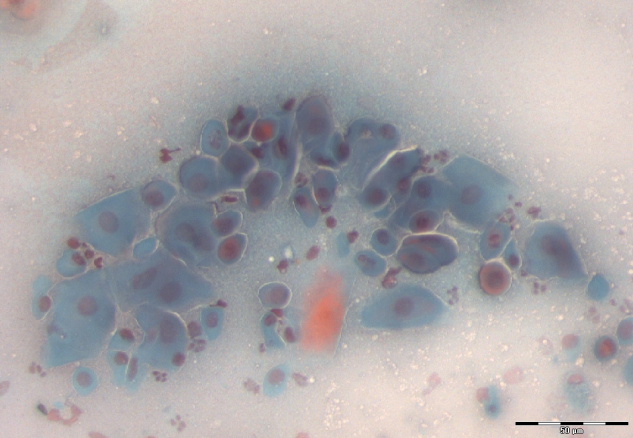

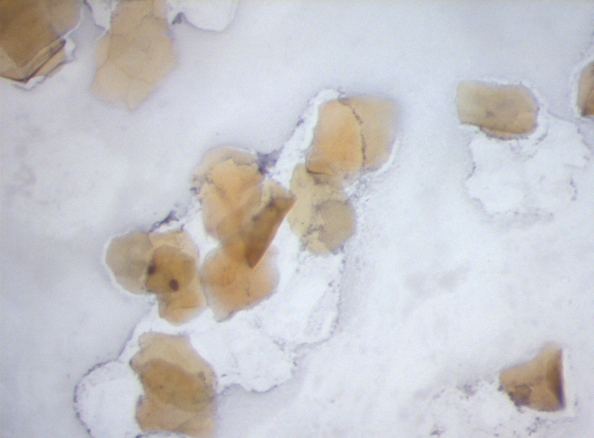

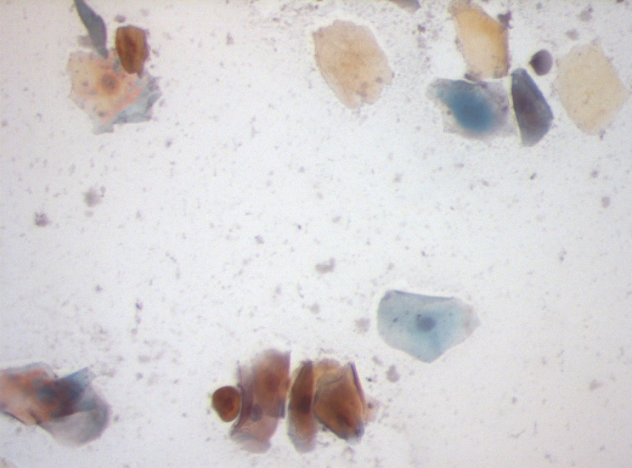

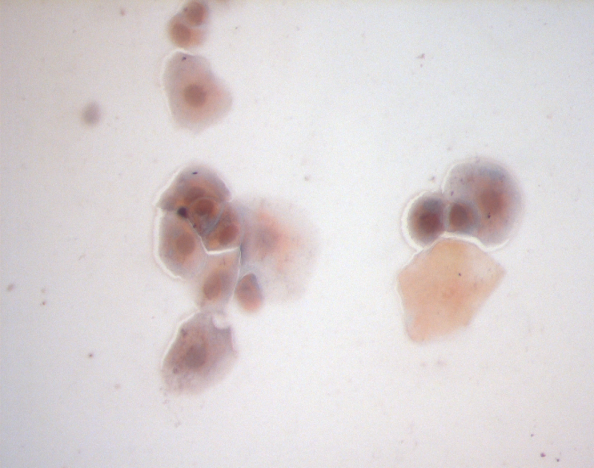

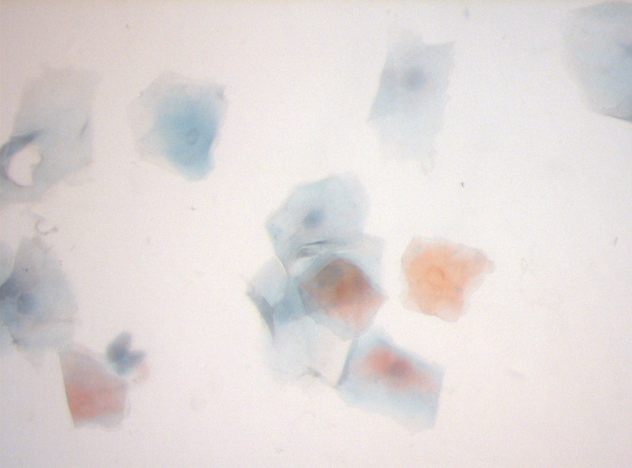


A B C

D E

A B
