# Supplementary material for: Physiologically Persistent Corpora lutea in Eurasian Lynx (Lynx lynx) – Longitudinal Ultrasound and Endocrine Examinations Intra-Vitam
Source: PLoS One. 2014 Mar 5;9(3):e90469. doi: 10.1371/journal.pone.0090469 (PMC3943960; doi:10.1371/journal.pone.0090469)
Supplement: Table S1 — Origins and reproductive status of study animals. The origins of the captive and free-ranging animals are listed below. Furthermore the reproductive period they have been examined is listed in the table. (DOC) [file pone.0090469.s002.doc]

**Table S1: Origins and reproductive status of study animals.** The origins of the captive and free-ranging animals are listed below. Furthermore the reproductive period they have been examined is listed in the table.

| ID | Origin | pro-estrus | estrus | met-estrus | pregnancy | lactation | prolonged di-estrus |
| --- | --- | --- | --- | --- | --- | --- | --- |
| 1 | DE/Stendal | 2012 |  |  | 2011, 2011 | 2011 | 2010, 2010 |
| 2 | DE/Thale |  |  |  |  |  | 2010, 2010 |
| 3 | DE/Johannismuehle |  |  |  |  |  | 2010, 2010, 2011, 2012 |
| 4 | DE/Stralsund | 2012 | 2011 |  |  |  | 2010, 2010 |
| 5 | DE/Bischofswerda | 2012 | 2011 |  |  |  | 2010, 2010, 2011 |
| 6 | DE/Bischofswerda | 2012 | 2011 |  |  |  | 2010, 2010, 2011 |
| 7 | DE/Essehof | 2011 |  |  | 2012 | 2010 | 2010, 2011 |
| 8 | DE/Magdeburg |  |  |  |  |  | 2010, 2011 |
| 9 | DE/Johannismuehle |  |  |  |  |  | 2010, 2011, 2012 |
| 10 | DE/Thale | 2012 |  | 2012 |  |  |  |
| S1 | NO/Scandlynx |  |  |  |  |  | 2012 |
| S2 | NO/Scandlynx | 2012 |  |  |  |  |  |
| S3 | NO/Scandlynx |  |  |  |  |  | 2011 |
| S4 | NO/Scandlynx | 2011 |  |  |  |  |  |
| S5 | NO/Scandlynx |  | 2011 |  |  |  |  |
| S6 | NO/Scandlynx | 2012 |  |  |  |  |  |
| S7 | NO/Scandlynx | 2012 |  |  |  |  |  |
| S8 | NO/Scandlynx |  |  | 2011 |  |  |  |
| S9 | NO/Scandlynx |  |  | 2011 |  |  |  |
| S10 | NO/Scandlynx |  |  |  |  |  | 2011 |
