# Supplementary material for: Physiologically Persistent Corpora lutea in Eurasian Lynx (Lynx lynx) – Longitudinal Ultrasound and Endocrine Examinations Intra-Vitam
Source: PLoS One. 2014 Mar 5;9(3):e90469. doi: 10.1371/journal.pone.0090469 (PMC3943960; doi:10.1371/journal.pone.0090469)
Supplement: Table S2 — Data sharing. (DOC) [file pone.0090469.s003.doc]

**Table S2: Data sharing**

| ID | age | Monat | VL | intraVL | VR | intraVR |
| --- | --- | --- | --- | --- | --- | --- |
| 7C | 6 | 1 | 0.21 | 0.05216667 | 0.18 | 0.112 |
| 4d | 5 | 1 | 0.17 | 0.04 | 0.07 | 0.05 |
| 1f | 11 | 2 | 0.22 | 0.10333333 | 0.22 | 0.076 |
| 10A | 2 | 2 | NA | NA | 0.08 | 0.05 |
| 6c | 11 | 3 | 0.31 | 0.11 | 0.26 | 0.112 |
| 6E | 12 | 3 | 0.23 | 0.1 | 0.25 | 0.1075 |
| 5c | 5 | 3 | 0.22 | 0.09 | 0.27 | 0.09666667 |
| 5E | 6 | 3 | 0.24 | 0.108 | 0.24 | 0.06 |
| 7E | 7 | 3 | 0.24 | 0.1225 | 0.3 | 0.124 |
| 1c | 11 | 3 | 0.25 | 0.10166667 | 0.26 | 0.135 |
| 4c | 4 | 3 | 0.14 | 0.05333333 | NA | NA |
| 10B | 2 | 3 | 0.3 | 0.065 | 0.11 | 0.0525 |
| 1d | 11 | 4 | 0.35 | 0.12166667 | 0.28 | 0.11666667 |
| 3e | 9 | 5 | 0.24 | 0.09 | 0.24 | 0.102 |
| 9c | 4 | 5 | 0.18 | 0.09 | 0.15 | 0.062 |
| 3A | 7 | 6 | 0.23 | 0.08833333 | 0.26 | 0.12166667 |
| 1A | 10 | 6 | 0.21 | 0.125 | 0.29 | 0.10428571 |
| 4A | 3 | 6 | 0.17 | 0.05 | 0.14 | 0.05 |
| 2A | 20 | 6 | 0.22 | 0.07 | 0.26 | 0.08 |
| 6A | 10 | 7 | 0.29 | 0.116 | 0.21 | 0.098 |
| 5A | 4 | 7 | 0.22 | 0.11333333 | 0.27 | 0.14 |
| 3d | 11 | 7 | 0.19 | 0.08333333 | 0.13 | 0.08833333 |
| 9B | 3 | 7 | NA | NA | NA | NA |
| 7A | 5 | 8 | 0.28 | 0.1 | 0.21 | 0.074014 |
| 1e | 11 | 8 | 0.26 | 0.11571429 | 0.26 | 0.10428571 |
| 6D | 11 | 10 | 0.28 | 0.096 | 0.21 | 0.076 |
| 5D | 6 | 10 | 0.15 | 0.07 | 0.19 | 0.0575 |
| 7D | 6 | 10 | 0.18 | NA | 0.15 | NA |
| 8B | 3 | 10 | 0.19 | 0.0975 | 0.21 | 0.086 |
| 7B | 5 | 11 | 0.2 | 0.09333333 | 0.22 | 0.09833333 |
| 1B | 10 | 11 | 0.24 | 0.11166667 | 0.27 | 0.09833333 |
| 6B | 11 | 12 | 0.23 | 0.096 | 0.17 | 0.08333333 |
| 5B | 4 | 12 | 0.15 | 0.06 | 0.2 | 0.076 |
| 3B | 7 | 12 | 0.13 | 0.064 | 0.14 | 0.062 |
| 9A | 2 | 12 | 0.19 | 0.06833333 | 0.12 | 0 |
| 8A | 2 | 12 | NA | NA | NA | NA |
| 4b | 3 | 12 | 0.24 | 0 | 0.18 | 0 |
| 2B | 20 | 12 | 0.12 | 0 | 0.14 | 0 |
| 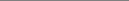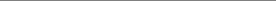   | 1 | | --- | | 3 | 2 | 0.2 | 0.102 | 0.22 | 0.11 |
| 2 | NA | 2 | 0.19 | 0.10666667 | 0.18 | 0.12 |
| S1w | 5 | 2 | NA | NA | NA | NA |
| S2w | 5 | 2 | NA | NA | NA | NA |
| S3w | 5 | 2 | NA | NA | NA | NA |
| 3 | NA | 2 | 0.23 | 0.115 | 0.19 | 0.1075 |
| 4 | NA | 2 | 0.26 | 0.106 | 0.2 | 0.1 |
| S4w | 5 | 3 | NA | NA | NA | NA |
| S5w | 12 | 3 | NA | NA | NA | NA |
| S6w | 4 | 3 | NA | NA | NA | NA |
|  |  |  |  |  |  |  |
|  |  |  |  |  |  |  |
| E2ng/ml | P4ng/ml | PGFMng/ml | volumeL | nfl | f1la | f2la |
| 0.12 | 6.51 | 2.26 | 3.1010614 | 0 | 0 | 0 |
| 0.37 | 2.02 | 1.39 | 0.08946816 | 0 | 0 | 0 |
| 1.16 | 10.03 | 2.12 | 1.14454504 | 0 | 0 | 0 |
| 1.51 | 1.58 | 0.91 | 0.11400211 | 1 | 0.19 | 0 |
| 0.28 | 1.85 | 2.63 | 4.0698783 | 2 | 0.95 | 0.72 |
| 0.55 | 2.32 | 2.57 | 6.07595407 | 0 | 0 | 0 |
| 0.17 | 7.34 | 1.77 | 2.529202 | 2 | 0.94 | 0.62 |
| 0.70 | 2.12 | 0.87 | 0.14476459 | 0 | 0 | 0 |
| 0.70 | 180.40 | 1.97 | 1.66046445 | 0 | 0 | 0 |
| 0.54 | 27.60 | 1.88 | 1.80973408 | 0 | 0 | 0 |
| 0.09 | 0.39 | 2.73 | 2.04448959 | 0 | 0 | 0 |
| 1.39 | 18.80 | 1.15 | 0.48366782 | 0 | 0 | 0 |
| 0.43 | 44.15 | 3.97 | 5.9711152 | 1 | NA | 0 |
| 0.39 | 4.20 | 2.3 | 1.50295756 | 2 | 0.3 | 0.28 |
| 1.11 | 3.68 | 2.01 | 1.04362451 | 1 | 0.27 | 0 |
| 0.27 | 3.28 | 1.16 | 1.61457442 | 0 | 0 | 0 |
| 0.38 | 6.06 | NA | 2.13226177 | 0 | 0 | 0 |
| 0.09 | 0.00 | 1.24 | 0.41430146 | 2 | 0.01 | 0.11 |
| 0.36 | 1.99 | 2.05 | 1.93622638 | 0 | 0 | 0 |
| 0.19 | 8.41 | 1.41 | 2.95890904 | 0 | 0 | 0 |
| 0.20 | 4.11 | 2.05 | 2.51660814 | 1 | 0.16 | 0 |
| 0.41 | 2.51 | 1.17 | 2.16260955 | 0 | 0 | 0 |
| 0.25 | 3.10 | 1.69 | 1.94344576 | 0 | 0 | 0 |
| 0.18 | 3.14 | 4.41 | 3.13279619 | 0 | 0 | 0 |
| 0.44 | 170.38 | 1.48 | 2.15317535 | 0 | 0 | 0 |
| 0.25 | 6.05 | 0.71 | 2.29029566 | 1 | 0.5 | 0 |
| 0.25 | 3.48 | 0.91 | 1.52791359 | 1 | 0.62 | 0 |
| 0.26 | 5.02 | 2.26 | 2.92003183 | 0 | 0 | 0 |
| 0.69 | 12.56 | NA | 1.54377863 | 0 | 0 | 0 |
| 0.18 | 9.06 | 1.90 | 3.53016997 | 0 | 0 | 0 |
| 0.35 | 5.46 | 2.87 | 2.59817802 | 0 | 0 | 0 |
| 0.07 | 13.66 | 0.95 | 2.30585047 | 1 | 0.35 | 0 |
| 0.29 | 6.24 | 2.16 | 0.8322668 | 0 | 0 | 0 |
| 0.24 | 3.10 | 1.02 | 1.28963635 | 0 | 0 | 0 |
| 0.16 | 0.00 | 1.43 | 0.59304159 | 0 | 0 | 0 |
| 0.35 | 8.36 | 4.73 | NA | 0 | 0 | 0 |
| 0.12 | 0.35 | 3.69 | 0.64284526 | 0 | 0 | 0 |
| 0.39 | 2.21 | 1.05 | 0.16681857 | 0 | 0 | 0 |
| 0.8 | 2.3 | 3.95 | 1.59985606 | 0 | 0 | 0 |
| 0.08 | 0.37 | 2.63 | 0.73944295 | 0 | 0 | 0 |
| 0.85 | 3.97 | 2.13 | 3.87235852 | 5 | NA | NA |
| 0.48 | 0.86 | 2.69 | 2.96138304 | 1 | NA | 0 |
| 1.46 | 2.57 | 1.55 | 2.00393399 | 0 | 0 | 0 |
| 0.45 | 1.59 | 1.21 | 1.71469384 | 0 | 0 | 0 |
| 0.87 | 2.31 | 3.53 | 2.72187588 | 0 | 0 | 0 |
| 0.49 | 3.25 | 1.77 | 2.94336758 | 0 | 0 | 0 |
| NA | NA |  | 2.62996544 | 1 | 0.54 | 0 |
| NA | NA |  | 2.82248538 | 1 | NA | 0 |
|  |  |  |  |  |  |  |
|  |  |  |  |  |  |  |

| f3la | f1lb | f2lb | f3lb | nclL | cl1la | cl2la |
| --- | --- | --- | --- | --- | --- | --- |
| 0 | 0 | 0 | 0 | 4 | 0.65 | 0.68 |
| 0 | 0 | 0 | 0 | 3 | 0.7 | 0.51 |
| 0 | 0 | 0 | 0 | 3 | 0.62 | 0.52 |
| 0 | 0.14 | 0 | 0 | 0 | 0 | 0 |
| 0 | 0.61 | 0.27 | 0 | 4 | 0.82 | 0.92 |
| 0 | 0 | 0 | 0 | 5 | 1.27 | 1.15 |
| 0 | 0.78 | 0.34 | 0 | 1 | 0.81 | 0 |
| 0 | 0 | 0 | 0 | 1 | 0.65 | 0 |
| 0 | 0 | 0 | 0 | 6 | 0.44 | 0.71 |
| 0 | 0 | 0 | 0 | 4 | 0.83 | 0.64 |
| 0 | 0 | 0 | 0 | 3 | 0.48 | 0.42 |
| 0 | 0 | 0 | 0 | 1 | 0.96 | 0 |
| 0 | NA | 0 | 0 | 3 | 0.74 | 1.17 |
| 0 | 0 | 0 | 0 | 3 | 0.43 | 0.6 |
| 0 | 0.12 | 0 | 0 | 2 | 0.7 | 0.54 |
| 0 | 0 | 0 | 0 | 2 | 0.64 | 0.74 |
| 0 | 0 | 0 | 0 | 2 | 0.69 | 0.81 |
| 0 | 0.01 | 0.11 | 0 | 1 | 0.59 | 0 |
| 0 | 0 | 0 | 0 | 0 | 0 | 0 |
| 0 | 0 | 0 | 0 | 3 | 0.7 | 0.6 |
| 0 | 0.16 | 0 | 0 | 2 | 0.74 | 0.68 |
| 0 | 0 | 0 | 0 | 4 | 0.41 | 0.35 |
| 0 | 0 | 0 | 0 | 2 | 1.09 | 1.28 |
| 0 | 0 | 0 | 0 | 3 | 1.16 | 0.85 |
| 0 | 0 | 0 | 0 | 3 | 0.81 | 0 |
| 0 | 0.32 | 0 | 0 | 4 | 0.64 | 0.51 |
| 0 | 0.3 | 0 | 0 | 4 | 0.76 | 0.76 |
| 0 | 0 | 0 | 0 | 4 | 0.72 | 0.87 |
| 0 | 0 | 0 | 0 | 3 | 0.73 | 0.56 |
| 0 | 0 | 0 | 0 | 4 | 0.73 | 0.63 |
| 0 | 0 | 0 | 0 | 3 | 0.66 | 0.64 |
| 0 | 0.21 | 0 | 0 | 4 | 0.41 | 0.42 |
| 0 | 0 | 0 | 0 | 2 | 0.42 | 0.63 |
| 0 | 0 | 0 | 0 | 2 | 0.53 | 0.47 |
| 0 | 0 | 0 | 0 | NA | 0 | 0 |
| 0 | 0 | 0 | 0 | NA | 0 | 0 |
| 0 | 0 | 0 | 0 | 1 | 0.44 | 0 |
| 0 | 0 | 0 | 0 | NA | 0 | 0 |
| 0 | 0 | 0 | 0 | 1 | 0.86 | 0 |
| 0 | 0 | 0 | 0 | 5 | 0.63 | 0.27 |
| NA | NA | NA | NA | 4 | 0.3 | 0.54 |
| 0 | NA | 0 | 0 | 4 | 0.65 | 0.65 |
| 0 | 0 | 0 | 0 | 4 | 0.69 | 0.46 |
| 0 | 0 | 0 | 0 | 3 | 0.78 | 0.48 |
| 0 | 0 | 0 | 0 | 5 | 0.8 | 0.48 |
| 0 | 0 | 0 | 0 | 6 | 0.54 | 0.37 |
| 0 | 0.75 | 0 | 0 | 5 | 0.88 | 0.67 |
| 0 | NA | 0 | 0 | 4 | 0.79 | 0.82 |
|  |  |  |  |  |  |  |
|  |  |  |  |  |  |  |

| cl3la | cl4la | cl5la | cl6la | cl1lb | cl2lb | cl3lb |
| --- | --- | --- | --- | --- | --- | --- |
| 0.72 | 0.65 | 0 | 0 | 0.64 | 0.5 | 0.43 |
| 0.64 | 0 | 0 | 0 | 0.39 | 0.46 | 0.55 |
| 0.56 | 0 | 0 | 0 | 0.33 | 0.34 | 0.37 |
| 0 | 0 | 0 | 0 | 0 | 0 | 0 |
| 0.59 | 0.52 | 0 | 0 | 0.64 | 0.49 | 0.67 |
| 0.45 | 0.51 | 0.58 | 0 | 1.21 | 0.85 | 0.38 |
| 0 | 0 | 0 | 0 | 0.5 | 0 | 0 |
| 0 | 0 | 0 | 0 | 0.37 | 0 | 0 |
| 0.8 | 0.5 | 0.53 | 0.71 | 0.51 | 0.56 | 0.72 |
| 0.64 | 0.55 | 0 | 0 | 0.41 | 0.41 | 0.51 |
| 0.61 | 0 | 0 | 0 | 0.41 | 0.51 | 0.58 |
| 0 | 0 | 0 | 0 | 0.79 | 0 | 0 |
| 0.48 | 0 | 0 | 0 | 0.8 | 0.92 | 0.55 |
| 0.47 |  |  |  | 0.5 | 0.27 | 0.38 |
|  |  |  |  | 0.51 | 0.46 |  |
| 0 | 0 | 0 | 0 | 1 | 0.41 | 0 |
| 0.58 | 0.61 | 0.56 | 0 | 0.36 | 0.57 | 0.43 |
| 0 | 0 | 0 | 0 | 0.44 | 0 | 0 |
| 0 | 0 | 0 | 0 | 0 | 0 | 0 |
| 0.57 | 0 | 0 | 0 | 0.7 | 0.6 | 0.57 |
| 0 | 0 | 0 | 0 | 0.79 | 0.77 | 0 |
| 0.62 | 0.77 | 0 | 0 | 0.32 | 0.38 | 0.49 |
| 0 | 0 | 0 | 0 | 0.62 | 0.53 | 0 |
| 0.86 | 0 | 0 | 0 | 0.951 | 0.49 | 0.69 |
| 0 | 0 | 0 | 0 | 1.23 | 0 | 0 |
| 0.6 | 0.46 | 0 | 0 | 0.44 | 0.47 | 0.62 |
| 0.55 | 0.49 | 0 | 0 | 0.67 | 0.63 | 0.74 |
| 0.6 | 0.61 | 0 | 0 | 0.67 | 0.83 | 0.58 |
| 0.66 | 0 | 0 | 0 | 0.46 | 0.54 | 0.66 |
| 0.53 | 0.77 | 0 | 0 | 0.67 | 0.59 | 0.71 |
| 0.5 | 0 | 0 | 0 | 0.66 | 0.39 | 0.47 |
| 0.56 | 0.63 | 0.58 | 0 | 0.52 | 0.58 | 0.54 |
| 0 | 0 | 0 | 0 | 0.43 | 0.53 | 0 |
| 0 | 0 | 0 | 0 | 0.6 | 0.35 | 0 |
| 0 | 0 | 0 | 0 | 0 | 0 | 0 |
| 0 | 0 | 0 | 0 | 0 | 0 | 0 |
| 0 | 0 | 0 | 0 | 0.53 | 0 | 0 |
| 0 | 0 | 0 | 0 | 0 | 0 | 0 |
| 0 | 0 | 0.00 | 0.00 | 0.65 | 0.00 | 0.00 |
| 0.41 | 0.39 | 0.65 | 0.00 | 0.41 | 0.25 | 0.39 |
| 1.07 | 0.65 | 0.00 | 0.00 | 0.39 | 0.54 | 0.70 |
| 0.55 | 0.64 | 0.00 | 0.00 | 0.65 | 0.82 | 0.48 |
| 0.47 | 0.76 | 0.00 | 0.00 | 0.64 | 0.42 | 0.45 |
| 0.67 | 0 | 0.00 | 0.00 | 0.75 | 0.59 | 0.69 |
| 0.66 | 0.79 | 0.55 | 0.00 | 0.66 | 0.81 | 0.75 |
| 0.36 | 0.36 | 0.88 | 1.20 | 0.54 | 0.40 | 0.41 |
| 0.77 | 0.84 | 0.67 | 0.00 | 0.57 | 0.33 | 0.49 |
| 0.97 | 0.68 | 0.00 | 0.00 | 0.39 | 0.56 | 0.62 |
|  |  |  |  |  |  |  |
|  |  |  |  |  |  |  |

| cl4lb | cl5lb | cl6lb | volareaL | volumeR | nfr | f1ra |
| --- | --- | --- | --- | --- | --- | --- |
| 0.32 | 0 | 0 | 1.0002831 | 0.72057297 | 0 | 0 |
| 0 | 0 | 0 | 0.67512826 | 1.80164684 | 0 | 0 |
| 0 | 0 | 0 | 0.46228536 | 2.95601092 | 2 | 0.33 |
| 0 | 0 | 0 | 0 | 0.36522742 | 4 | 0.37 |
| 0.56 | 0 | 0 | 1.30541029 | 3.5023653 | 2 | 0.85 |
| 0.43 | 0.35 | 0 | 2.44062479 | 2.78950887 | 4 | 0.8 |
| 0 | 0 | 0 | 0.31808626 | 2.63120951 | 3 | 0.43 |
| 0 | 0 | 0 | 0.18888826 | 1.18531663 | 0 | 0 |
| 0.26 | 0.35 | 0.34 | 1.37829524 | 6.29123564 | 0 | 0 |
| 0.34 | 0 | 0 | 0.87658289 | 1.57699233 | 0 | 0 |
| 0 | 0 | 0 | 0.60067252 | 0.62404047 | 0 | 0 |
| 0 | 0 | 0 | 0.59564597 | 0.32656856 | 0 | 0 |
| 0 | 0 | 0 | 1.51770341 | 5.4679483 | 3 | NA |
|  |  |  | 0.43636722 | 0.85809776 | 2 | 0.27 |
|  |  |  | 0.47548005 | 1.29974364 | 1 | 0.34 |
| 0 | 0 | 0 | 0.74094463 | 1.84853197 | 1 | 0.52 |
| 0.63 | 0.53 | 0 | 1.28852423 | 2.2455476 | 0 | 0 |
| 0 | 0 | 0 | 0.20388936 | 0.27597242 | 0 | 0 |
| 0 | 0 | 0 | 0 | 3.57052529 | 3 | 0.21 |
| 0 | 0 | 0 | 0.9227643 | 4.05836594 | 1 | 0.12 |
| 0 | 0 | 0 | 0.87037824 | 1.79063241 | 0 | 0 |
| 0.66 | 0 | 0 | 0.8452455 | 1.82551823 | 0 | 0 |
| 0 | 0 | 0 | 1.06358619 | 0.13410831 | 0 | 0 |
| 0 | 0 | 0 | 1.65959344 | 1.08212944 | 0 | 0 |
| 0 | 0 | 0 | 0.78249219 | 3.05061213 | 0 | 0 |
| 0.48 | 0 | 0 | 0.87501209 | 3.46046038 | 3 | 0.54 |
| 0.41 | 0 | 0 | 1.25341693 | 2.9788935 | 0 | 0 |
| 0.67 | 0 | 0 | 1.54032288 | 1.79348498 | 0 | 0 |
| 0 | 0 | 0 | 0.84336055 | 1.1545353 | 0 | 0 |
| 0.77 | 0 | 0 | 1.43727864 | 2.26521083 | 0 | 0 |
| 0 | 0 | 0 | 0.72272339 | 1.37320978 | 0 | 0 |
| 0.61 | 0.51 |  | 1.13042358 | 3.34668917 | 0 | 0 |
| 0 | 0 | 0 | 0.40408736 | 0.92245014 | 0 | 0 |
| 0 | 0 | 0 | 0.37895461 | 0.65463565 | 0 | 0 |
| 0 | 0 | 0 | 0 | 0.13995795 | 0 | 0 |
| 0 | 0 | 0 | 0 | NA | 0 | 0 |
| 0 | 0 | 0 | 0.18315485 | 1.33002231 | 0 | 0 |
| 0 | 0 | 0 | 0 | 0.34636059 | 0 | 0 |
| 0.00 | 0.00 |  |  | 1.43 | 0.00 | 0.00 |
| 0.38 | 0.36 |  |  | 2.38 | 3.00 | 0.67 |
| 0.39 | 0.00 | 0.00 |  | 0.45 | 0.00 | 0.00 |
| 0.55 | 0.00 | 0.00 |  | 2.24 | 0.00 | 0.00 |
| 0.71 | 0.00 | 0.00 |  | 1.97 | 1.00 | NA |
| 0.00 | 0.00 | 0.00 |  | 1.35 | 0.00 | 0.00 |
| 0.43 | 0.81 | 0.00 |  | 0.68 | 10.00 | NA |
| 0.37 | 0.62 | 0.81 |  | 2.70 | 0.00 | 0.00 |
| 0.54 | 0.46 | 0.00 |  | 2.23 | 4.00 | 0.42 |
| 0.71 | 0.00 | 0.00 |  | 2.81 | 0.00 | 0.00 |
|  |  |  |  |  |  |  |
|  |  |  |  |  |  |  |
|  |  |  |  |  |  |  |

| f2ra | f3ra | f4ra | f1rb | f2rb | f3rb | f4rb |
| --- | --- | --- | --- | --- | --- | --- |
| 0 | 0 | 0 | 0 | 0 | 0 | 0 |
| 0 | 0 | 0 | 0 | 0 | 0 | 0 |
| 0.31 | 0 | 0 | 0.16 | 0.15 | 0 | 0 |
| 0.36 | 0.15 | 0.37 | 0.22 | 0.24 | 0.14 | 0.25 |
| 0.28 | 0 | 0 | 0.63 | 0.36 | 0 | 0 |
| 0.54 | 0.47 | 0.46 | 0.61 | 0.24 | 0.21 | 0.25 |
| 0.62 | 0 | 0 | 0.65 | 0.32 | 0 | 0 |
| 0 | 0 | 0 | 0 | 0 | 0 | 0 |
| 0 | 0 | 0 | 0 | 0 | 0 | 0 |
| 0 | 0 | 0 | 0 | 0 | 0 | 0 |
| 0 | 0 | 0 | 0 | 0 | 0 | 0 |
| 0 | 0 | 0 | 0 | 0 | 0 | 0 |
| NA | NA | 0 | NA | NA | NA | 0 |
| 0.42 | 0 | 0 | 0.1 | 0.12 | 0 | 0 |
| 0 | 0 | 0 | 0.22 | 0 | 0 | 0 |
| 0 | 0 | 0 | 0.59 | 0 | 0 | 0 |
| 0 | 0 | 0 | 0 | 0 | 0 | 0 |
| 0 | 0 | 0 | 0 | 0 | 0 | 0 |
| 0.17 | 0.12 | 0 | 0.21 | 0.17 | 0.12 | 0 |
| 0 | 0 | 0 | 0.2 |  | 0 | 0 |
| 0 | 0 | 0 | 0 | 0 | 0 | 0 |
| 0 | 0 | 0 | 0 | 0 | 0 | 0 |
| 0 | 0 | 0 | 0 | 0 | 0 | 0 |
| 0 | 0 | 0 | 0 | 0 | 0 | 0 |
| 0 | 0 | 0 | 0 | 0 | 0 | 0 |
| 0.16 | 0.1 | 0 | 0.29 | 0.16 | 0.1 | 0 |
| 0 | 0 | 0 | 0 | 0 | 0 | 0 |
| 0 | 0 | 0 | 0 | 0 | 0 | 0 |
| 0 | 0 | 0 | 0 | 0 | 0 | 0 |
| 0 | 0 | 0 | 0 | 0 | 0 | 0 |
| 0 | 0 | 0 | 0 | 0 | 0 | 0 |
| 0 | 0 | 0 | 0 | 0 | 0 | 0 |
| 0 | 0 | 0 | 0 | 0 | 0 | 0 |
| 0 | 0 | 0 | 0 | 0 | 0 | 0 |
| 0 | 0 | 0 | 0 | 0 | 0 | 0 |
| 0 | 0 | 0 | 0 | 0 | 0 | 0 |
| 0 | 0 | 0 | 0 | 0 | 0 | 0 |
| 0 | 0 | 0 | 0 | 0 | 0 | 0 |
| 0.00 | 0.00 | 0 | 0 | 0 | 0 | 0 |
|  | 0.00 | 0 | 0.32 |  | 0 | 0 |
| 0.00 | 0.00 | 0 | 0 | 0 | 0 | 0 |
| 0.00 | 0.00 | 0 | 0 | 0 | 0 | 0 |
| 0.00 | 0.00 | 0 | NA | 0 | 0 | 0 |
| 0.00 | 0.00 | 0 | 0 | 0 | 0 | 0 |
| NA | NA | NA | NA | NA | NA | NA |
| 0.00 | 0.00 | 0 | 0 | 0 | 0 | 0 |
| 0.54 | 0.00 | 0 | 0.78 | 0.45 | 0 | 0 |
| 0.00 | 0.00 | 0 | 0 | 0 | 0 | 0 |
|  |  |  |  |  |  |  |
|  |  |  |  |  |  |  |

| nclr | cl1ra | cl2ra | cl3rb | cl4ra | cl5ra | cl6ra |
| --- | --- | --- | --- | --- | --- | --- |
| 2 | 0.65 | 0.55 | 0 | 0 | 0 | 0 |
| 3 | 0.4 | 0.62 | 0.44 | 0 | 0 | 0 |
| 4 | 0.88 | 0.72 | 0.74 | 0.47 | 0 | 0 |
| 0 | 0 | 0 | 0 | 0 | 0 | 0 |
| 2 | 0.51 | 0.67 | 0 | 0 | 0 | 0 |
| 4 | 0.71 | 0.83 | 0.8 | 0.85 | 0 | 0 |
| 1 | 0.57 | 0 | 0 | 0 | 0 | 0 |
| 1 | 0.47 | 0 | 0 | 0 | 0 | 0 |
| 5 | 0.7 | 0.6 | 1.37 | 0.77 | 0.94 |  |
| 3 | 1 | 0.7 | 0.94 | 0 | 0 | 0 |
| NA |  |  |  | 0 | 0 | 0 |
| 0 | 0 | 0 | 0 | 0 | 0 |  |
| 4 | 0.69 | 1.16 | 0.56 | 0.55 | 0 | 0 |
| 2 | 0.73 | 0.72 | 0 | 0 | 0 | 0 |
| 3 | 0.64 | 0.83 | 0.65 | 0 | 0 | 0 |
| 1 | 0.68 | 0 | 0 | 0 | 0 | 0 |
| 3 | 0.55 | 0.42 | 0.58 | 0 | 0 | 0 |
| 0 | 0 | 0 | 0 | 0 | 0 | 0 |
| 1 | 0.67 | 0 | 0 | 0 | 0 | 0 |
| 3 | 1.05 | 0.65 | 0.59 | 0 | 0 | 0 |
| 3 | 0.5 | 0.58 | 0.78 | 0 | 0 | 0 |
| 3 | 0.57 | 0.46 | 0.64 | 0 | 0 | 0 |
| 0 | 0 | 0 | 0 | 0 | 0 | 0 |
| 3 | 0.87 | 1.06 | 0.95 | 0.98 | 0 | 0 |
| 2 | 0.77 | 0.98 | 0 | 0 | 0 | 0 |
| 5 | 0.75 | 0.57 | 0.76 | 0.79 | 0.59 | 0 |
| 3 | 0.87 | 0.56 | 0.56 | 0 | 0 | 0 |
| 5 | 0.68 | 0.56 | 0.61 | 0.74 | 0.69 | 0 |
| 1 | 0.55 | 0 | 0 | 0 | 0 | 0 |
| 2 | 0.75 | 0.63 | 0 | 0 | 0 | 0 |
| 3 | 0.82 | 0.62 | 0.64 | 0 | 0 | 0 |
| 6 | 0.43 | 0.25 | 0.38 | 0.54 | 0.59 | 0.54 |
| 3 | 0.48 | 0.41 | 0.5 | 0 | 0 | 0 |
| 2 | 0.64 | 0.37 | 0 | 0 | 0 | 0 |
| NA | 0 | 0 | 0 | 0 | 0 | 0 |
| NA | 0 | 0 | 0 | 0 | 0 | 0 |
| 0 |  |  |  | 0 | 0 | 0 |
| NA | 0 | 0 | 0 | 0 | 0 | 0 |
| 2 | 0.74 | 0.94 | 0 | 0 | 0 | 0 |
| 7 | 0.37 | 0.56 | 0.56 | 0.82 | 0.8 | 0.4 |
| 4 | 0.43 | 0.33 | 0.37 | 0.36 | 0 | 0 |
| 1 | 0.59 | 0 | 0 | 0 | 0 | 0 |
| 2 | 0.96 | 0.93 | 0 | 0 | 0 | 0 |
| 3 | 0.75 | 0.57 | 0.81 | 0 | 0 | 0 |
| 2 | 0.64 | 0.97 | 0 | 0 | 0 | 0 |
| NA | 0 | 0 | 0 | 0 | 0 | 0 |
| 4 | 0.7 | 0.82 | 0.71 | 0.7 | 0 | 0 |
| 6 | 0.75 | 0.94 | 0.65 | 0.92 | 0.69 | 0.91 |
|  |  |  |  |  |  |  |
|  |  |  |  |  |  |  |

| cl6ra | cl1rb | cl2rb | cl3b | cl4rb | cl5rb | cl6rb |
| --- | --- | --- | --- | --- | --- | --- |
| 0 | 0.71 | 0.67 | 0 | 0 | 0 | 0 |
| 0 | 0.5 | 0.42 | 0.51 | 0 | 0 | 0 |
| 0 | 0.53 | 0.3 | 0.44 | 0.26 | 0 | 0 |
| 0 | 0 | 0 | 0 | 0 | 0 | 0 |
| 0 | 0.61 | 0.58 | 0 | 0 | 0 | 0 |
| 0 | 0.59 | 0.61 | 0.47 | 0.53 | 0 | 0 |
| 0 | 0.42 | 0 | 0 | 0 | 0 | 0 |
| 0 | 0.82 | 0 | 0 | 0 | 0 | 0 |
|  | 0.62 | 0.41 | 0.89 | 1.37 | 0.64 |  |
| 0 | 0.69 | 0.56 | 0.44 | 0 | 0 | 0 |
| 0 | 0 | 0 | 0 | 0 | 0 | 0 |
| 0 | 0 | 0 | 0 | 0 | 0 | 0 |
| 0 | 0.6 | 0.83 | 0.51 | 0.57 | 0 | 0 |
| 0 | 0.42 | 0.65 | 0 | 0 | 0 | 0 |
| 0 | 0.42 | 0.58 | 0.57 | 0 | 0 | 0 |
| 0 | 0.59 | 0 | 0 | 0 | 0 | 0 |
| 0 | 0.4 | 0.45 | 0.51 | 0 | 0 | 0 |
| 0 | 0 | 0 | 0 | 0 | 0 | 0 |
| 0 | 0.52 | 0 | 0 | 0 | 0 | 0 |
| 0 | 0.57 | 0.56 | 0.68 | 0 | 0 | 0 |
| 0 | 0.5 | 0.52 | 0.7 | 0 | 0 | 0 |
| 0 | 0.8 | 0.65 | 0.63 | 0 | 0 | 0 |
| 0 | 0 | 0 | 0 | 0 | 0 | 0 |
| 0 | 0.99 | 0.89 | 1.27 | 0.74 | 0 | 0 |
| 0 | 1.06 | 0.82 | 0 | 0 | 0 | 0 |
| 0 | 0.32 | 0.42 | 0.52 | 0.47 | 0.5 | 0 |
| 0 | 0.73 | 0.68 | 0.57 | 0 | 0 | 0 |
| 0 | 0.6 | 0.53 | 0.8 | 0.58 | 0.6 | 0 |
| 0 | 0.55 | 0 | 0 | 0 | 0 | 0 |
| 0 | 0.6 | 0.42 | 0 | 0 | 0 | 0 |
| 0 | 0.62 | 0.82 | 0 | 0 | 0 | 0 |
| 0 | 0.41 | 0.42 | 0.47 | 0.61 | 0.4 | 0.49 |
| 0 | 0.54 | 0.51 | 0.5 | 0 | 0 | 0 |
| 0 | 0.54 | 0.41 | 0 | 0 | 0 | 0 |
| 0 | 0 | 0 | 0 | 0 | 0 | 0 |
| 0 | 0 | 0 | 0 | 0 | 0 | 0 |
| 0 | 0 | 0 | 0 | 0 | 0 | 0 |
| 0 | 0 | 0 | 0 | 0 | 0 | 0 |
| 0 | 0.72 | 0.72 | 0 | 0 | 0 | 0 |
| 0.64 | 0.6 | 0.76 | 0.82 | 0.55 | 0.7 | 0.38 |
| 0 | 0.41 | 0.34 | 0.42 | 0.33 | 0 | 0 |
| 0 | 0.48 | 0 | 0 | 0 | 0 | 0 |
| 0 | 0.75 | 0.56 | 0 | 0 | 0 | 0 |
| 0 | 0.56 | 0.79 | 0.63 | 0 | 0 | 0 |
| 0 | 0.31 | 0.72 | 0 | 0 | 0 | 0 |
| 0 | 0 | 0 | 0 | 0 | 0 | 0 |
| 0 | 0.57 | 0.38 | 0.52 | 0.54 | 0 | 0 |
| 0 | 0.79 | 0.98 | 0.59 | 0.74 | 0.63 | 0.72 |
|  |  |  |  |  |  |  |
|  |  |  |  |  |  |  |

| cl7rb | volareaR |
| --- | --- |
| 0 | 0.65188048 |
| 0 | 0.53784066 |
| 0 | 0.887657 |
| 0 | 0 |
| 0 | 0.54954309 |
| 0 | 1.37578196 |
| 0 | 0.18802432 |
| 0 | 0.30269245 |
|  | 2.79271879 |
| 0 | 1.17464149 |
| 0 | 0 |
| 0 | 0 |
| 0 | 1.55186823 |
| 0 | 0.60836942 |
| 0 | 0.88019572 |
| 0 | 0.31510174 |
| 0 | 0.55354863 |
| 0 | 0 |
| 0 | 0.27363272 |
| 0 | 1.07104748 |
| 0 | 0.86205302 |
| 0 | 0.90964815 |
| 0 | 0 |
| 0 | 2.9345617 |
| 0 | 1.27218795 |
| 0 | 1.21022003 |
| 0 | 1.04858509 |
| 0 | 1.59907066 |
| 0 | 0.23758294 |
| 0 | 0.56124553 |
| 0 | 0.79859285 |
| 0 | 1.01308509 |
| 0 | 0.5641515 |
| 0 | 0.39057851 |
| 0 | 0 |
| 0 | 0 |
| 0 | 0 |
| 0 | 0 |
| 0 |  |
| 0.46 |  |
| 0 |  |
| 0 |  |
| 0 |  |
| 0 |  |
| 0 |  |
| 0 |  |
| 0 |  |
| 0 |  |
|  |  |
|  |  |
