# Supplementary material for: Physiologically Persistent Corpora lutea in Eurasian Lynx (Lynx lynx) – Longitudinal Ultrasound and Endocrine Examinations Intra-Vitam
Source: PLoS One. 2014 Mar 5;9(3):e90469. doi: 10.1371/journal.pone.0090469 (PMC3943960; doi:10.1371/journal.pone.0090469)
Supplement: Table S3 — Comparison between free-ranging and captive lynx. Comparison of various ovarian and serum parameters between single examinations of free-ranging (N = 10) and captive lynx (N = 10) during February and March 2011 and 2012. (DOCX) [file pone.0090469.s004.docx]

**Table S3: Comparison between free-ranging and captive lynx.** Comparison of various ovarian and serum parameters between single examinations of free-ranging (N = 10) and captive lynx (N = 10) during February and March 2011 and 2012.

|  | **P4** | **E2** | **PGFM** | **diameter** | **ovarian** | **number** | **CL** | **age in** |
| --- | --- | --- | --- | --- | --- | --- | --- | --- |
|  |  |  |  | ***a.ovarica*** | **volume** | **CL** | **tissue** | **years** |
| **W - value** | 30 |  |  | 105 | 172 |  | 129 | 112.5 |
| **t** |  | 0.11 | -1.42 |  |  | -2.17 |  |  |
| **p-value** | 0.86 | 0.91 | 0.18 | 0.07 | 0.46 | 0.04 | 0.06 | 0.05 |
