# Supplementary material for: Physiologically Persistent Corpora lutea in Eurasian Lynx (Lynx lynx) – Longitudinal Ultrasound and Endocrine Examinations Intra-Vitam
Source: PLoS One. 2014 Mar 5;9(3):e90469. doi: 10.1371/journal.pone.0090469 (PMC3943960; doi:10.1371/journal.pone.0090469)
Supplement: Table S4 — Repeated measurements from six captive Eurasian lynx throughout one reproductive year (February – February). Measurements were categorized in 3 time periods, excluding estrus (pro-estrus, pseudo-pregnancy, prolonged di-estrus) and tested if there are seasonal changes within an individual. (DOC) [file pone.0090469.s005.doc]

**Table S4: Repeated measurements from six captive Eurasian lynx throughout one reproductive year (February – February).** Measurements were categorized in 3 time periods, excluding estrus (pro-estrus, pseudo-pregnancy, prolonged di-estrus) and tested if there are seasonal changes within an individual.

| **Quade** | **P4** | **E2** | **PGFM** | **diameter** | **ovarian** | **number** | **CL tissue** | **amount** |
| --- | --- | --- | --- | --- | --- | --- | --- | --- |
| **test** |  |  |  | **A.ovarica** | **volume** | **CL** |  | **follicles** |
| **F-value** | 0.07 | 2.26 | 0.02 | 0.88 | 0.21 | 0.61 | 0.83 | 0.08 |
| **df** | 22 | 22 | 22 | 22 | 22 | 22 | 22 | 22 |
| **p-value** | 0.93 | 0.13 | 0.98 | 0.43 | 0.81 | 0.55 | 0.45 | 0.92 |
